# Enhanced performance of an eco-friendly and sustainable secalin-corn starch packaging film via citric acid cross-linking

**DOI:** 10.1016/j.crfs.2025.101231

**Published:** 2025-10-24

**Authors:** Sona Dodange, Hajar Shekarchizadeh

**Affiliations:** Department of Food Science and Technology, College of Agriculture, Isfahan University of Technology, Isfahan, 84156-83111, Iran

**Keywords:** Secalin, Citric acid, Cross-linking, Food packaging, Mushroom

## Abstract

Growing concerns over plastic pollution have intensified the search for sustainable food packaging solutions. This study introduces a novel biodegradable film based on secalin (SCL) protein from rye, a rarely explored biopolymer, blended with corn starch (CS) and cross-linked with citric acid (CA). Films were prepared at different SCL/CS ratios (1:2, 1:1, 2:1) and CA concentrations (0 %, 2 %, 5 %, 10 %) and comprehensively characterized. Field emission-scanning electron microscopy (FE-SEM) and thickness analysis confirmed that CA enhanced phase compatibility, while barrier tests revealed improved water vapor and oxygen resistance through CA-induced tortuous diffusion paths. The optimized 2:1 SCL/CS film with 2 % CA exhibited balanced mechanical strength, barrier performance, and strong antioxidant/antimicrobial activities, attributed to metal ion chelation and bacterial membrane disruption. Unlike conventional biodegradable films, this formulation was successfully applied as active packaging for mushrooms, showing improved preservation by reducing weight loss and visual spoilage under ambient conditions. These findings highlight the timely potential of SCL-based composites as multifunctional, eco-friendly packaging materials for perishable foods.

## Highlights

• Cross-linkers enhance the performance of biopolymer-based packaging.
• Citric acid cross-linked corn starch/secalin films improved packaging properties.
• SCL/CS/CA films extended mushroom shelf life as active packaging.
• Citric acid boosted antibacterial, antioxidant, and barrier properties in films.

## Graphical abstract

**Figure:**
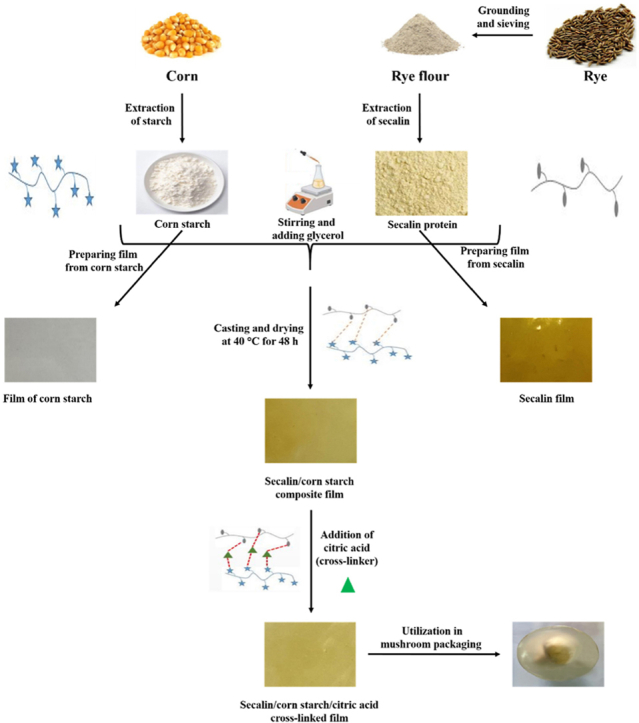

## 1 Introduction

Food packaging is crucial because it must protect products from microbiological, chemical, and physical damage throughout production, storage, and consumption, thereby maintaining quality and preventing deterioration (Jiang et al., 2023a; Jiang et al., 2023b). Plastic-based packaging is commonly used in the food industry due to its excellent mechanical and barrier properties, as well as cost-effectiveness. However, in recent years, concerns have grown regarding pollution from non-degradable petroleum-based packaging and the migration of harmful chemicals into packaged food. This has led to increased attention on biopolymer-based materials, such as proteins and polysaccharides (Wen et al., 2021). Proteins and polysaccharides offer several advantages, including abundance, availability, biodegradability, eco-friendliness, and low cost (Wang et al., 2023a). The main limitation hindering the widespread adoption of biodegradable packaging is its weak mechanical and barrier properties. Recently, researchers have explored strategies to overcome these limitations, such as developing blend films, incorporating nanomaterials, designing multilayer packaging, and applying cross-linkers (Wei et al., 2024).

Cereals are a major natural source of protein, and the extracted proteins can be used to prepare biodegradable films. The primary storage proteins in cereals are prolamins, which are alcohol-soluble. Secalin (SCL) is the prolamin protein of rye (*Secale cereale* L.), and its main amino acids are proline and glutamine (Qazanfarzadeh et al., 2021a). Recently, SCL has been investigated as a biopolymer for food packaging. Like other protein films, however, it suffers from hydrophilicity and generally exhibits weak mechanical properties and brittleness (Qazanfarzadeh et al., 2021b). To address these drawbacks, this study used corn starch (CS) as a co-polymer and citric acid (CA) as a cross-linker. CS, a polysaccharide extracted from corn, has several advantageous properties, including biodegradability, good film-forming ability, and oxygen barrier properties. Nevertheless, CS-based films also present some limitations, such as low mechanical strength, limited hydrophobicity, and poor thermal stability (Srivastava et al., 2023). Previous studies have developed blend films using protein-starch combinations, including gelatin/CS (Susmitha et al., 2021; Kumar et al., 2021), zein/starch (Li et al., 2021; Masanabo et al., 2022), soy protein isolate/starch (Cheng et al., 2025), and whey protein isolate/starch (Zheng et al., 2024).

Cross-linking is an effective method to reduce hydrophilicity while improving the mechanical and barrier properties of polymer-based films. This process links polymer chains via covalent or non-covalent bonds, forming a three-dimensional network that restricts polymer mobility and enhances film performance. Citric acid (CA, C_6_H_8_O_7_) is a tricarboxylic acid and a green additive in the food industry. It is water-soluble and naturally found in various organisms, vegetables, and fruits, particularly citrus fruits such as lemons (Zhang et al., 2023). CA has been widely applied as a plasticizer and/or cross-linking agent in film preparation. In recent years, its use as a cross-linker has received considerable attention (Wen et al., 2021; Yao et al., 2022; Wang et al., 2022, 2023b). However, previous studies using CA have mostly focused on common proteins such as gelatin, zein, or soy protein, while little attention has been given to secalin (SCL), the main storage protein of rye. SCL has a distinct amino acid composition (rich in proline and glutamine) and relatively higher hydrophobicity compared to other prolamins, which can enhance compatibility with starch and potentially improve barrier and functional properties. Unlike the extensively studied proteins, SCL therefore provides a novel basis for designing composite films with unique characteristics.

Moreover, most existing reports have emphasized only the improvement of mechanical and barrier properties, whereas other critical challenges in the adoption of sustainable packaging—such as scalability, cost-effectiveness, and compliance with food safety regulations—remain underexplored. Addressing these aspects is essential to evaluate the real-world potential of biodegradable films.

In critically reviewing the literature, it can be observed that gelatin/CS or zein/starch films improved flexibility but often lacked strong antioxidant activity (Susmitha et al., 2021; Kumar et al., 2021; Li et al., 2021; Masanabo et al., 2022), while soy or whey protein–starch blends enhanced barrier properties but showed limited antimicrobial functions (Cheng et al., 2025; Zheng et al., 2024). In contrast, our approach leverages the hydrophobic nature of SCL together with the film-forming ability of CS and the cross-linking effect of CA to obtain multifunctional films with superior barrier, antioxidant, and antimicrobial properties. To the best of our knowledge, this is the first systematic study on SCL/CS blends cross-linked with CA, thereby advancing the development of novel sustainable food packaging materials.

In this study, to address the limitations of pure SCL films, we investigated the combined effects of CS and CA. Blend films with different SCL/CS ratios were prepared, and various concentrations of CA were incorporated as a chemical cross-linker to formulate the cross-linked films. The effects of both blending and cross-linking on the films were systematically evaluated. The prepared films were characterized for a range of properties, including barrier performance against oxygen and water vapor, mechanical strength, microstructure, thermal stability, chemical interactions, as well as antioxidant and antibacterial activities. The results clearly demonstrated how the addition of CS and CA influenced the overall properties of SCL films. Finally, the selected cross-linked film was practically tested as active packaging for mushrooms, demonstrating its functional advantages and potential applicability in preserving the quality of perishable foods.

## 2 Materials and methods

### 2.1 Materials

Rye, mushrooms, and sunflower oil were purchased from a local market in Isfahan, Iran. Chemical reagents, including corn starch, sodium metabisulfite, citric acid, glycerol, activated carbon, sodium chloride (NaCl), calcium chloride (CaCl_2_), reduced iron powder, and ethanol, were obtained from Merck KGaA Co. (Darmstadt, Germany).

### 2.2 Extraction of secalin protein from rye seeds

Rye seeds were first cleaned, ground, and sieved through a 60-mesh sieve to obtain uniform flour. Secalin (SCL) protein was extracted following the method of Qazanfarzadeh et al. (2021a) with slight modifications. Briefly, rye flour was mixed with 70 % (v/v) ethanol at a 1:5 (w/v) ratio, along with 0.5 % (w/w) sodium metabisulfite, and stirred at 50 °C for 1 h. The mixture was then centrifuged at 7000×*g* for 5 min (Sigma, Germany). The resulting supernatant was added to cold distilled water (4 °C) to precipitate secalin. The precipitated protein was collected by centrifugation at 7000×*g* and 4 °C for 5 min, freeze-dried for 24 h (Sanat Pardaz Dena Co., Iran), and defatted using hexane at a 1:10 (w/v) ratio. The obtained SCL was stored at 4 °C until further use.

### 2.3 Film preparation based on SCL and CS

For SCL solution preparation, 2 g of the extracted protein was dissolved in 100 mL of 70 % (v/v) ethanol. The suspension pH was adjusted to 9.0 using 1N NaOH, and glycerol (40 % w/w) was added as a plasticizer. The solution was stirred at 50 °C for 1 h (Qazanfarzadeh et al., 2021b). Simultaneously, a corn starch (CS) solution was prepared by dissolving 2 g of CS in 100 mL of distilled water, adding glycerol (40 % w/w), and stirring in a 90 °C water bath for 1 h (Kumar et al., 2021). SCL and CS solutions were mixed in different ratios (1:1, 1:2, and 2:1) at 50 °C for 30 min. For cross-linked films, citric acid (CA) was added at 2, 5, or 10 % (w/w) and stirred at 50 °C for 30 min. Then, 40 mL of each prepared solution was poured into 90-mm plastic petri dishes and oven-dried at 40 °C for 48 h (Memmert, Germany). The dried films were peeled off and conditioned in a desiccator at 55 % relative humidity. Pure CS and SCL films were also prepared as control films.

The chosen SCL/CS ratios and CA concentrations were selected based on ranges reported in previous studies as effective for protein–starch blends and citric acid cross-linking, thereby enabling systematic evaluation of compositional effects on film properties (Qazanfarzadeh et al., 2021b; Kumar et al., 2021).

### 2.4 Characterization of prepared films based on SCL and CS

#### 2.4.1 Thickness, moisture content, color, and opacity

A digital micrometer (Mitutoyo-Co, Japan) with a sensitivity of 0.001 mm was used to measure the thickness of the prepared films at ten different points, including the center and corners of each film.

Moisture content (MC) was determined by measuring the weight difference of 1.5 × 1.5 cm^2^ pieces of each film before (W_0_) and after (W_1_) oven-drying at 105 °C for 24 h (Wang et al., 2023b), using Eq. (1).

(1) $$Moisture\;content\;\left(\%\right)=\frac{\left(W_{0}-W_{1}\right)}{W_{0}}\times 100$$

The color parameters of the prepared films—whiteness/darkness (L∗), redness/greenness (a∗), and blueness/yellowness (b∗)—were measured using a colorimeter (Nippon Denshoku, Japan).

Opacity was determined by measuring the absorbance of 1.5 × 4 cm^2^ film pieces with a UV–Vis spectrophotometer (Union, USA) at 600 nm (Yao et al., 2022), using Eq. (2):

(2) $$Opacity\;\left({mm}^{-1}\right)=\frac{A_{600}}{x}$$

where A_600_ is the absorbance of the film at 600 nm and x is the mean thickness (mm) of the film.

#### 2.4.2 Permeability to water vapor and oxygen

Water vapor permeability (WVP) of the prepared films was determined by attaching the films to the 8-mm holes in the lids of cups containing 3 g of dried CaCl_2_. The cups were placed in a desiccator containing distilled water at 100 % relative humidity and 25 °C. The cups were weighed every 24 h for 7 days (Dodange et al., 2024a). WVP was calculated using Eq. (3), where WVTR, X, A, and ΔP represent the rate of water vapor permeation (g/s), the mean film thickness (m), the hole area of the cup lids (m^2^), and the pressure difference between the dried CaCl_2_ and the desiccator environment (Pa), respectively.

(3) $$Water\;Vapor\;permeability\;\left(g.m/Pa.s.m^{2}\right)=\frac{WVTR\times X}{A\times \Delta P}$$

Oxygen permeability (O_2_P) was measured by covering the lids of cups with the prepared films and filling the cups with a deoxidizing agent mixture containing 1.5 g NaCl, 1 g activated carbon, and 0.5 g reduced iron powder. The cups were then placed in a desiccator containing distilled water at 100 % relative humidity and 25 °C for 48 h (Yang et al., 2021). O_2_P was calculated using Eq. (4), where m_f_, m_i_, t, and A represent the weight of the cups after 48 h (g), the initial weight of the cups (g), the test duration (s), and the effective area of the films (m^2^), respectively.

(4) $$Oxygen\;permeability\;\left(g/s.m^{2}\right)=\frac{m_{f}-m_{i}}{t\times A}$$

#### 2.4.3 Mechanical properties

Tensile strength (TS) and elongation at break (EAB) of the prepared films were measured using a Universal Testing Machine (Bongshin, Korea). For this, 1.5 × 4 cm film pieces were placed between the grips of the device (Wang et al., 2023b). The testing speed, grip distance, and maximum tensile load were set to 50 mm/min, 40 mm, and 20 kN, respectively. TS and EAB were calculated using Eqs. (5), (6)):

(5) $$Tensile\;strength\;\left(MPa\right)=\frac{F_{\max}}{A}$$

(6) $$Elongation\;at\;break\;\left(\%\right)=\frac{L_{\max}}{L_{0}}\times 100$$

where F_max_, A, L_max_ and L_0_ represent the maximum force (N), the cross-sectional area of the film (m^2^), the length at rupture (m), and the initial length of the film (m), respectively.

#### 2.4.4 Antioxidant and antimicrobial activities

To determine the antioxidant activity of the prepared SCL, CS, SCL/CS composite, and SCL/CS/CA cross-linked films, 25 mg of each sample was soaked in 4 mL of a 100 μM ethanolic DPPH solution and incubated for 30 min at 25 °C in the dark. The absorbance of each solution was then measured at 517 nm using a UV–Vis spectrophotometer (Maroufi et al., 2021). The antioxidant activity was calculated using Eq. (7):

(7) $$DPPH\;radical\;scavenging\;activity\;\left(\%\right)=\frac{A_{DPPH}-A_{s}}{A_{DPPH}}\times 100$$

Herem A_DPPH_ and A_S_ represent the absorbance of the DPPH solution and the sample, respectively.

The antibacterial activity of SCL, CS, SCL/CS, and SCL/CS/CA films was evaluated using gram-negative (*E. coli*) and gram-positive (*S. aureus*) bacteria via the agar disk diffusion method. Petri dishes containing nutrient agar were cultured with 10^5^ CFU/mL of the bacteria. Due to the poor diffusivity of solid films in agar, aqueous solutions of SCL, CS, SCL/CS, and SCL/CS/CA films were used instead of intact films. These solutions were added into 5 mm diameter wells in the agar. The plates were incubated at 37 °C for 24 h, and inhibition zones, indicating areas where bacterial growth was prevented, were measured using a ruler (Chowdhury et al., 2020).

Also, minimum inhibitory concentration (MIC) and minimum bactericidal concentration (MBC) tests were performed as additional antimicrobial assays to determine the antibacterial activity of citric acid against *E. coli* and *S. aureus*. For the MIC test, 100 μL of citric acid solution at various concentrations ranging from 3200 to 1.5625 μg/mL was prepared through serial dilutions and added to a 96-well plate containing 95 μL of nutrient broth and 5 μL of bacterial suspension. The first well in which bacterial growth was not visibly observed and appeared clear was selected as the MIC after 18–24 h of incubation at 37 °C. The MBC was determined by subculturing the wells on agar plates, where no visible bacterial growth was observed after 18–24 h of incubation at 37 °C. In other words, the MBC was defined as the lowest concentration at which all bacteria were killed (Chowdhury et al., 2020).

#### 2.4.5 CA release

The release of CA from the prepared film into three types of food simulants—sunflower oil, ethanol (50 % v/v), and distilled water—over a period of 5 days was measured. For this, a 1.5 × 1.5 cm^2^ piece of the selected cross-linked film was immersed in 15 mL of each simulant. The tests were conducted under static conditions (without agitation), with the simulants kept in closed glass vials at 25 °C. To ensure accurate quantification, calibration curves were constructed separately in sunflower oil, distilled water, and ethanol (50 % v/v) by measuring the absorbance of CA standard solutions at different concentrations at 210 nm. The corresponding linear regression equations were used to calculate the concentration of CA released from the films. Measurements were performed using a UV–Vis spectrophotometer at a wavelength of 210 nm on the 1st, 3rd, and 5th days (Dodange et al., 2024b).

#### 2.4.6 Self-healing property

To study the self-healing property of the SCL/CS/CA cross-linked film, a controlled scratch (∼2 cm width) was created on the surface of the film using a sterile scalpel. Then, 50 μL of distilled water was carefully injected onto the scratched area at 25 °C. The healing process was monitored under an optical microscope (Olympus Optical Co., Japan), and images were captured at 0 min (immediately after scratching) and 10 min. The degree of healing was evaluated by comparing the reduction in scratch width over time. Furthermore, to determine the self-healing efficiency of the cross-linked film, the ratio of the tensile strength (TS) of the self-healed film to that of the original film was calculated (Liu et al., 2017).

#### 2.4.7 Water contact angle

The surface hydrophobicity/hydrophilicity of the films was evaluated by placing a 2 μL water droplet on 1.5 × 1.5 cm^2^ film samples using a syringe. The angle formed between the water droplet and the film surface was recorded as the water contact angle (WCA).

#### 2.4.8 Morphology

The surface properties of the prepared films were examined using a field emission-scanning electron microscope (FE-SEM, Zeiss, Germany). Before imaging, the film surfaces were coated with a thin gold layer using a sputter coater. The FE-SEM was operated at a magnification of × 500 and an accelerating voltage of 20 kV.

#### 2.4.9 Chemical structure

Interactions between the polymers and the cross-linker, as well as the functional groups present in the films, were analyzed using attenuated total reflectance Fourier transform infrared (ATR-FTIR) spectroscopy (Jasco, Japan). The spectra were recorded over a wavenumber range of 600–4000 cm^−1^.

#### 2.4.10 Thermal stability

Thermal stability of the prepared films was evaluated using a thermal analyzer (Netzsch, Germany). For each test, 5 mg of film was placed in an aluminum pan, and the temperature was increased from 25 to 480 °C at a rate of 10 °C/min under a nitrogen atmosphere.

### 2.5 Mushroom packaging

The potential of the prepared cross-linked films for food packaging was evaluated using fresh mushrooms. The effects of packaging on mushroom properties, including weight loss and appearance changes, were studied under three conditions: unsealed dishes, dishes sealed with polyethylene (PE), and dishes sealed with the cross-linked films. Unsealed samples served as controls to assess the impact of packaging, while PE, a commercial packaging material, was used as a benchmark. Experiments were conducted under controlled laboratory conditions (25 °C and 43 % relative humidity) for 5 days. These ambient conditions were chosen to accelerate spoilage for a clearer evaluation of the film's performance and to reflect typical retail and household storage practices, where mushrooms are often kept at room temperature.

### 2.6 Statistical analysis

The effects of different SCL/CS ratios and the addition of CA on film properties were analyzed using a completely randomized design in SAS 9.4 software. Significant differences among means were determined by analysis of variance (ANOVA) and further compared using Duncan's multiple range test (*p* < 0.05). All results are reported as mean ± standard deviation.

## 3 Results and discussions

### 3.1 Thickness, moisture content, color parameters and opacity

The list and appearance of the various designed films are presented in Table 1.

**Table 1:** Components, thickness and appearance of the various prepared films.

Name of films	SCL (g)	CS (g)	CA (% w/w)	Thickness (mm)	Appearance
SCL	0.80	–	–	0.168 ± 0.021^bcd^	
CS	–	0.80	–	0.168 ± 0.017^bcd^	
SCL/CS_1:1_	0.40	0.40	–	0.270 ± 0.040^a^	
SCL/CS_1:2_	0.27	0.53	–	0.273 ± 0.039^a^	
SCL/CS_2:1_	0.53	0.27	–	0.270 ± 0.008^a^	
SCL/CS_1:1_/CA_2 %_	0.40	0.40	2.00	0.149 ± 0.006^cd^	
SCL/CS_1:2_/CA_2 %_	0.27	0.53	2.00	0.149 ± 0.009^cd^	
SCL/CS_2:1_/CA_2 %_	0.53	0.27	2.00	0.148 ± 0.010^d^	
SCL/CS_1:1_/CA_5 %_	0.40	0.40	5.00	0.161 ± 0.010^cd^	
SCL/CS_1:2_/CA_5 %_	0.27	0.53	5.00	0.167 ± 0.005^bcd^	
SCL/CS_2:1_/CA_5 %_	0.53	0.27	5.00	0.169 ± 0.005^bc^	
SCL/CS_1:1_/CA_10 %_	0.40	0.40	10.00	0.182 ± 0.023^b^	
SCL/CS_1:2_/CA_10 %_	0.27	0.53	10.00	0.185 ± 0.010^b^	
SCL/CS_2:1_/CA_10 %_	0.53	0.27	10.00	0.185 ± 0.011^b^	

Various letters in column of thickness show significant differences between prepared films (*p* < 0.05).

Film thickness, an important physical property, influences several characteristics of the prepared films, including water vapor permeability (WVP), mechanical properties, and opacity. Thickness is affected by factors such as the molecular arrangement within the film, interactions between the film molecules and the solvent, and the chemical composition of the components (Long et al., 2024). The thickness values of the prepared films are summarized in Table 1. A significant difference (*p* < 0.05) was observed between the thickness of the SCL/CS composite films (0.270–0.273 mm) and the other films. The SCL/CS composite films were the thickest, likely due to their nonhomogeneous and rough structure, as also observed in FE-SEM images. Incorporation of CA into the SCL/CS/CA cross-linked films improved compatibility among the film components, resulting in a more compact structure and a significant reduction in thickness (Bhat et al., 2021). However, increasing the CA concentration slightly increased the thickness of the cross-linked films, likely due to the higher solid content in the film matrix. Similar trends were reported by Yao et al. (2022), who observed that adding higher concentrations of CA increased the thickness of mung bean starch films.

**Fig. 1:**
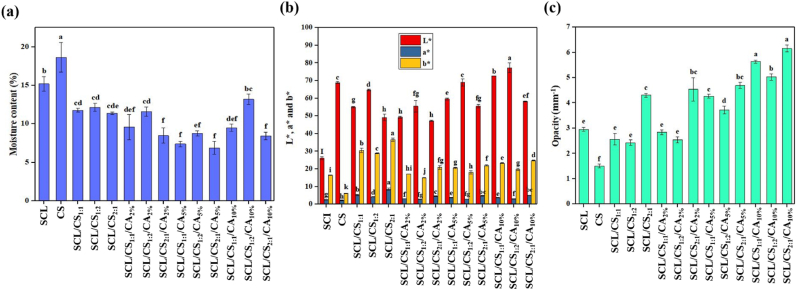
Moisture content (a), color parameters (b) and opacity (c) of different films (SCL: secalin, CS: corn starch, CA: citric acid, SCL/CS: composite films based on secalin and corn starch, and SCL/CS/CA: cross-linked film based on secalin, corn starch, and citric acid). Different letters denote significant differences at p < 0.05.

Studying the moisture content of the prepared films is important because it reflects their stability under varying humidity conditions. The moisture content of the different films is shown in Fig. 1a. The results indicated that the SCL/CS composite films (11.37–12.14 %) had significantly lower moisture content (*p* < 0.05) than the pure CS (18.63 %) and SCL (15.18 %) films. Incorporation of 5 % CA into the SCL/CS/CA cross-linked films further decreased the moisture content (6.90–11.59 %, *p* < 0.05). However, at a higher CA concentration of 10 %, the moisture content increased (8.45–13.18 %). The decrease in moisture at lower CA levels is attributed to hydrogen bonding between the carboxyl and hydroxyl groups of CA and the amino and hydroxyl groups of the film components. This interaction reduces the number of free hydrophilic groups in the films, limiting water absorption. At higher CA concentrations, unreacted hydroxyl groups in CA can interact with water molecules, forming additional hydrogen bonds, which increases moisture uptake in the cross-linked films (Martins et al., 2021). Wang et al. (2023b) reported similar findings for chitosan/ovotransferrin films with CA as a cross-linker.

The color of the films affects both consumer acceptance and the appearance of packaged food products (Zheng et al., 2019). Color measurements were recorded as L∗, a∗, and b∗ values (Fig. 1b). Higher L∗ values indicate brighter and more colorless films. Positive and negative a∗ values represent redness and greenness, while positive and negative b∗ values indicate yellowness and blueness. CS films exhibited the highest L∗ value (68.88) and the lowest a∗ and b∗ values (2.13 and 6.22, respectively), reflecting their colorless nature. Addition of SCL, a light-yellow protein (Qazanfarzadeh et al., 2021a), decreased L∗ (47.26–77.30) but increased a∗ (2.81–8.53) and b∗ (15.01–36.64) values. Thus, incorporating SCL reduced the lightness of CS films. Furthermore, SCL/CS composite and SCL/CS/CA cross-linked films showed yellow/red hues. The decrease in L∗ upon CA addition is likely due to electrostatic interactions and hydrogen bonding within the film matrix, which reduce film lightness (Wang et al., 2019). Similar observations were reported for chitosan/ovotransferrin films after CA cross-linking (Wang et al., 2023b).

The opacity of the prepared films is presented in Fig. 1c. The SCL/CS composite and SCL/CS/CA cross-linked films (2.43–6.16) showed significantly higher opacity (*p* < 0.05) than pure CS films (1.50). The increased opacity with higher CA concentrations is attributed to the formation of a more compact matrix in the cross-linked films, resulting from interactions among film components. This compact structure limits light transmission, helping to protect packaged foods from a wide range of radiation wavelengths. This higher opacity can be particularly advantageous for packaging light-sensitive foods, such as mushrooms, oils, or dairy products, where reduced light transmission helps slow down oxidative and photo-degradation reactions. Similar results were reported by Bhat et al. (2021) who observed an increase in the opacity of polyvinyl alcohol/guar gum/chitosan films after the addition of hydroxy citric acid as a cross-linker.

### 3.2 Permeability to water vapor and oxygen

**Fig. 2:**
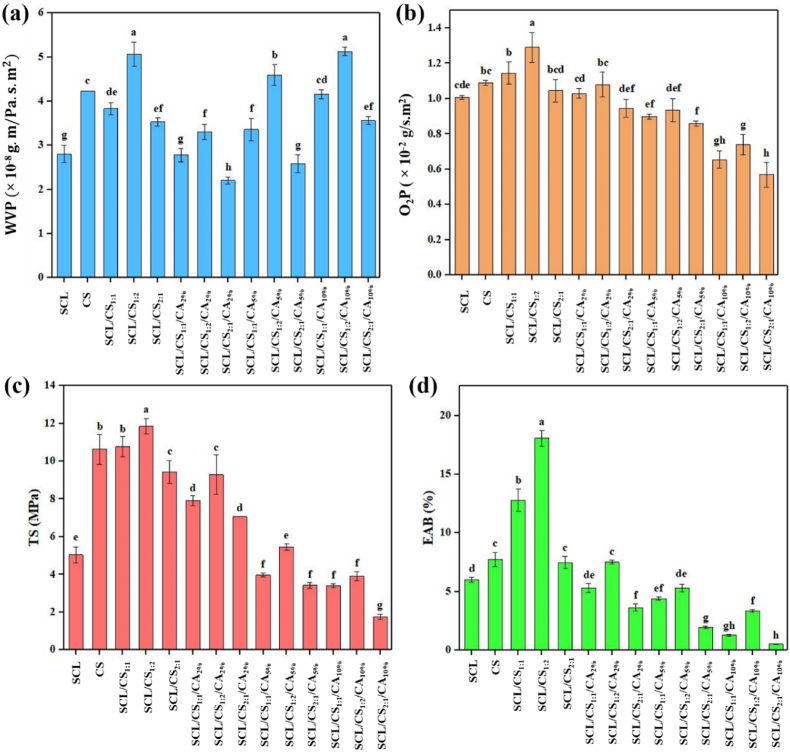
Water vapor permeability (a), oxygen permeability (b), tensile strength (c), and elongation at break (d) of different films. (SCL: secalin, CS: corn starch, CA: citric acid, SCL/CS: composite films based on secalin and corn starch, and SCL/CS/CA: cross-linked film based on secalin, corn starch, and citric acid). Different letters denote significant differences at p < 0.05.

Barrier properties against water molecules and gases such as O_2_ are among the most important characteristics of food packaging materials. Water vapor permeability (WVP) indicates the rate at which water vapor passes through a packaging film. The WVP of the prepared films is shown in Fig. 2a. CS exhibited higher WVP than SCL due to its many hydrophilic groups, such as –OH groups. Consequently, composite films with higher CS content also showed higher WVP. As illustrated in Fig. 2a, adding 2 % CA as a cross-linker significantly (*p* < 0.05) reduced WVP. This is attributed to the formation of a compact matrix through interactions among SCL, CS, and CA, which extends the diffusion path of water vapor. Furthermore, these interactions reduce the number of free amino and hydroxyl groups in SCL and CS, limiting their hydrogen bonding with water molecules. At higher CA concentrations, however, WVP increased. This is because excess CA introduces unreacted hydroxyl groups in the film, which interact with water vapor, leading to higher WVP in the SCL/CS/CA cross-linked films (Martins et al., 2021). Similar trends were reported by Wang et al. (2023b) for chitosan/ovotransferrin films with high CA content.

The oxygen permeability (O_2_P) of the prepared SCL, CS, SCL/CS composite, and SCL/CS/CA cross-linked films is shown in Fig. 2b. The O_2_P of the cross-linked films (0.57–1.08 × 10^−2^ g/s·m^2^) was significantly lower (*p* < 0.05) than that of the SCL/CS composite films (1.04–1.29 × 10^−2^ g/s·m^2^). The incorporation of CA reduces O_2_P by occupying pathways in the film matrix, forcing oxygen to follow a more tortuous route. This effect is more pronounced at higher CA concentrations, resulting in improved oxygen barrier properties in the cross-linked films. For comparison, conventional packaging materials commonly used in the food industry, such as polystyrene (PS), polyethylene (PE), and polypropylene (PP), exhibit O_2_P values of approximately 1.2–1.8 × 10^−2^, 0.6–2.4 × 10^−2^, and 0.6–1.2 × 10^−2^ g/s·m^2^, respectively (Michiels et al., 2017), indicating that the prepared SCL/CS/CA films have comparable or lower oxygen permeability and may offer superior barrier performance for food packaging applications.

### 3.3 Mechanical properties

Studying the mechanical properties of films is important because food packaging must remain intact until consumption and protect food from external forces during distribution (Liu et al., 2024). Two main factors affecting mechanical properties are the type of polymer and the interactions between film components. As shown in Fig. 2c and d, pure SCL films exhibited lower tensile strength (TS) and elongation at break (EAB) than CS films, likely due to the lower purity of extracted SCL compared to commercial CS. Similar results were reported by Qazanfarzadeh et al. (2021b), who observed lower TS for SCL films compared to zein films. The reduction in EAB of cross-linked films compared to blend films is explained by CA improving interactions between SCL and CS chains, producing a more compact structure and reducing stretchability (Wang et al., 2024). TS of cross-linked films also decreased relative to composite films, likely due to changes in polymer orientation induced by CA (Ahammed et al., 2021). Excessive cross-linker content can further reduce TS by creating a rigid film structure (Ghorpade et al., 2019; Diop et al., 2023).

Based on its acceptable mechanical and barrier properties, the 2:1 SCL/CS film containing 2 % CA was selected as the optimal cross-linked film for subsequent testing alongside its corresponding composite film.

### 3.4 Antioxidant and antimicrobial properties of films

**Fig. 3:**
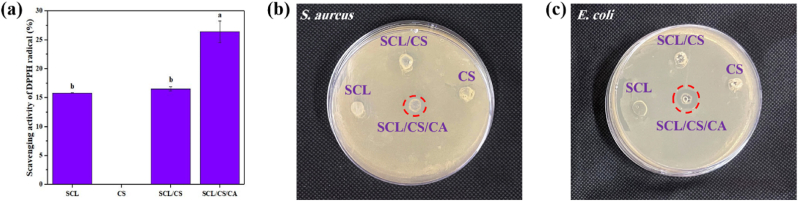
Antioxidant (a) and antimicrobial activities against *S. aureus* (b) and *E. coli* (c) of films. (SCL: secalin, CS: corn starch, CA: citric acid, SCL/CS: composite films based on secalin and corn starch, and SCL/CS/CA: cross-linked film based on secalin, corn starch, and citric acid). Different letters denote significant differences at p < 0.05.

Antioxidant packaging can prevent the oxidation of food components, particularly lipids, thus extending shelf life. The antioxidant activity of the films is shown in Fig. 3a. Pure CS film exhibited negligible activity, whereas SCL film demonstrated DPPH radical scavenging, possibly due to polyphenols present in the extracted protein. Similar findings were reported for kafirin, the prolamin protein from sorghum (Giteru et al., 2017). The presence of CA significantly (*p* < 0.05) increased the antioxidant activity of SCL/CS/CA cross-linked films. This effect is attributed to CA's ability to chelate metal ions (Pažarauskaitė et al., 2023), highlighting the potential of the cross-linked films for packaging oxidation-sensitive foods. CA can act as a natural antioxidant and synergize with other antioxidants (Azevedo et al., 2018).

The antimicrobial activity of the film solutions against Gram-positive (*S. aureus*) and Gram-negative (*E. coli*) bacteria is presented in Fig. 3b and c. SCL, CS, and SCL/CS composite solutions did not exhibit notable antimicrobial effects. In contrast, the SCL/CS/CA cross-linked film solution showed mild antibacterial activity, with inhibition zones of 7.14 ± 0.12 mm for *S. aureus* and 9.25 ± 0.31 mm for *E. coli*. CA exerts its antibacterial effect through two mechanisms: it increases bacterial cell membrane permeability, causing cytoplasmic leakage and cell death (Wang et al., 2022), and it chelates metal ions essential for bacterial growth. Although Gram-positive bacteria are generally more sensitive, the cross-linked film solution was more effective against E. coli, likely due to the higher density of chelating charges on Gram-negative cell surfaces. Similar effects of CA as a cross-linker were reported in chickpea, chitosan, and curcumin films (Yildiz et al., 2022).

It should also be noted that although aqueous film solutions were used in agar disk diffusion assays to improve diffusivity, this method still has inherent limitations. Therefore, to complement these results and provide a more accurate assessment, broth-based methods including MIC and MBC tests were performed. The MIC/MBC values of citric acid were 200/400 μg/mL for *E. coli* and 400/3200 μg/mL for *S. aureus*. These findings indicate that, as previously reported, *E. coli*, due to the chelating charges on its surface, exhibited greater sensitivity to citric acid compared to *S. aureus*.

### 3.5 CA release

**Fig. 4:**
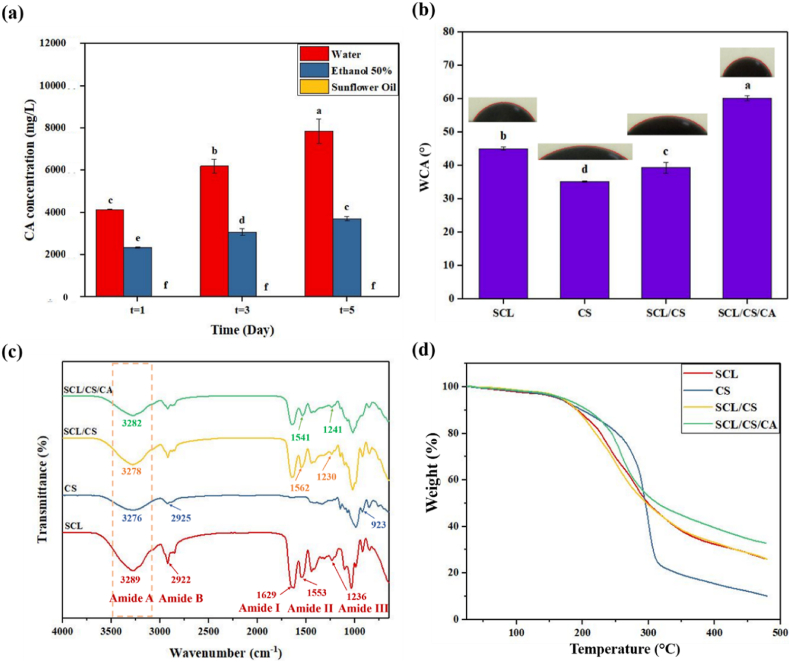
CA release from SCL/CS/CA film (a), water contact angle (WCA) (b), attenuated total reflectance Fourier transform infrared (c) and thermal gravimetric analysis (d) of films. (SCL: secalin, CS: corn starch, CA: citric acid, SCL/CS: composite films based on secalin and corn starch, and SCL/CS/CA: cross-linked film based on secalin, corn starch, and citric acid). Different letters denote significant differences at p < 0.05.

The migration behavior of packaging components was studied using food simulants: sunflower oil, ethanol (50 % v/v), and distilled water. The results of CA release are shown in Fig. 4a. CA release increased over time, and the type of food simulant had a significant effect (*p* < 0.05) on the release. Polarity of the food simulant is a key factor affecting CA release. Both SCL and CS contain many polar groups; therefore, polar simulants can penetrate the film matrix and dissolve the active compound. Among the tested simulants, water, being the most polar, penetrated the film matrix most effectively, leading to higher CA release. In contrast, sunflower oil, a non-polar simulant, showed negligible CA release. Ethanol (50 % v/v) exhibited intermediate behavior due to its lower polarity compared to water (Dodange et al., 2024b).

The release of CA from the SCL/CS/CA cross-linked film can contribute to preventing food oxidation and microbial spoilage due to its antioxidant and antibacterial activities. Similarly, Wei et al. (2024) reported higher release of procyanidins in 20 % ethanol than in 50 % ethanol due to differences in simulant polarity.

### 3.6 WCA

The wettability and hydrophobicity of the film surfaces were evaluated using WCA measurements, and results are shown in Fig. 4b. WCA is influenced by factors such as chemical composition, surface roughness, and microstructure (Ahammed et al., 2020). The pure CS film showed the lowest WCA (35.22°), indicating a highly hydrophilic surface, consistent with its high moisture content and WVP values. The SCL film exhibited a WCA of 45.05°. Its hydrophilicity may result from the use of 70 % ethanol during protein extraction, which reduces α-helix structures and increases β-sheet structures (Qazanfarzadeh et al., 2021b). The addition of CA significantly (*p* < 0.05) increased the WCA from 39.37° in the SCL/CS composite film to 60.19° in the SCL/CS/CA cross-linked film. This decrease in surface hydrophilicity is due to hydrogen bonding between the carboxyl and hydroxyl groups of CA and the amino and hydroxyl groups in the polymers. These interactions reduce the number of available hydroxyl groups and limit hydrogen bonding with water molecules (Bhat et al., 2021). Furthermore, the cross-linked films exhibited a denser structure, which also contributes to the higher WCA. Liu et al. (2024) similarly reported that CA addition to soybean polysaccharide films transformed the surface from hydrophilic to hydrophobic.

### 3.7 Chemical structure

The ATR-FTIR spectra of the prepared SCL, CS, SCL/CS composite, and SCL/CS/CA cross-linked films are shown in Fig. 4c. The characteristic bands of SCL at 3289, 2922, 1629, 1553, and 1236 cm^−1^ correspond to amide A, amide B, amide I, amide II, and amide III, representing the stretching vibration of N–H and O–H bonds, symmetric and asymmetric stretching of C–H, stretching of C=O, N–H bending, and N–H/C–N vibrations, respectively (Wang et al., 2023b). For CS, the main bands appeared at 3276, 2925, and 923 cm^−1^, associated with O–H stretching, C–H stretching in amylose, and the anhydroglucose ring stretching, respectively (Goiana et al., 2021).

When SCL and CS were blended, some band shifts occurred. Amide A shifted from 3289 to 3278 cm^−1^, indicating hydrogen bond formation between SCL amino groups and CS hydroxyl groups. Amide II and III shifted from 1553 to 1236 cm^−1^ to 1562 and 1230 cm^−1^, suggesting electrostatic interactions between SCL and CS. The addition of CA as a cross-linker further shifted amide A from 3278 to 3282 cm^−1^, confirming hydrogen bonding between the polymers and CA's carboxyl/hydroxyl groups. Amide II and III shifted from 1562 to 1230 cm^−1^ to 1541 and 1241 cm^−1^, indicating electrostatic interactions and hydrogen bonding between CA and the polymer matrix. These results confirm successful interaction of the cross-linker with the film polymers (Zhang et al., 2021). Similar observations were reported by Wang et al. (2023b) for chitosan/ovotransferrin films cross-linked with CA.

### 3.8 Thermal analysis

The TGA curves of the prepared films are presented in Fig. 4d. All samples exhibited three stages of weight loss. The first step (∼100 °C) corresponds to water evaporation. The second step (∼200–300 °C) is associated with plasticizer evaporation. The final step (∼300–500 °C) corresponds to polymer degradation. Residual weights for CS, SCL, SCL/CS, and SCL/CS/CA films were 10.14, 25.97, 26.19, and 32.88 %, respectively. The increased stability of the SCL/CS composite film is attributed to hydrogen bonding between SCL and CS. Furthermore, CA improved thermal stability in the SCL/CS/CA cross-linked film through ionic and hydrogen bonding with SCL and CS. Wang et al. (2022) reported similar improvements in thermal stability for nanocellulose/ɛ-poly-L-lysine aerogels cross-linked with CA.

### 3.9 Morphology

**Fig. 5:**
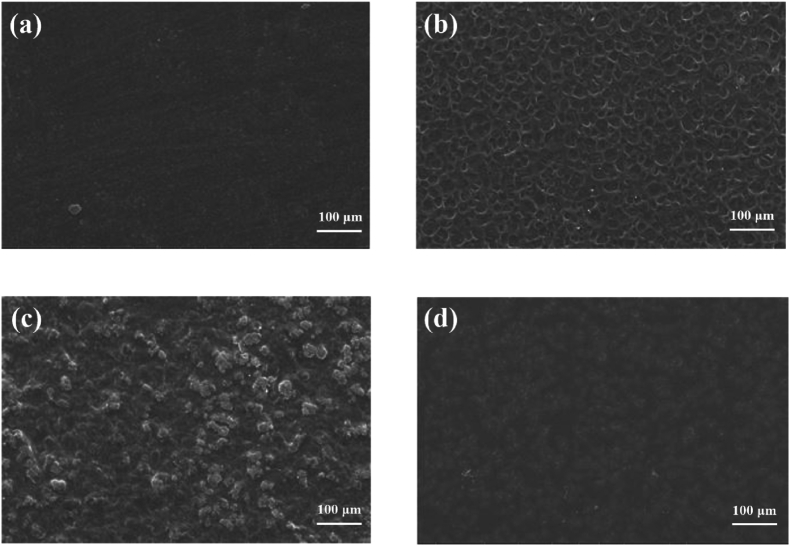
Field emission-scanning electron microscope images of SCL (a), CS (b), SCL/CS (c) and SCL/CS/CA (d) films. (SCL: secalin, CS: corn starch, CA: citric acid, SCL/CS: composite films based on secalin and corn starch, and SCL/CS/CA: cross-linked film based on secalin, corn starch, and citric acid).

The obtained results from the FE-SEM test are reported in Fig. 5. As can be seen, the surface of the SCL film appears smooth, with only a few small particles present on the surface of the film, which indicates good solubility of SCL in its solvent (70 % v/v ethanol) (Qazanfarzadeh et al., 2021b). The starch film, due to its granular structure, showed a rough surface (with measured hole dimensions of 30.8 ± 4.3 μm using ImageJ software) (Erna et al., 2022). The most heterogeneous and rough structure was observed in the SCL/CS composite film (with particle sizes of 18.4 ± 1.7 μm), which can contribute to its lower mechanical and barrier properties. Roughness on the film surface can act as stress points, facilitating scratch formation and reducing resistance to mechanical deformation, thereby negatively affecting barrier performance. Similar results were observed in SEM images of blended films based on soy protein isolate and oxidized corn starch (Liu et al., 2021). The SCL/CS/CA cross-linked film showed the smoothest and most homogeneous surface compared to the other films, which indicates excellent compatibility and the formation of interactions between SCL and CS as polymers, with CA as a cross-linker. The incorporation of CA enhanced film uniformity via electrostatic and hydrogen bonding with SCL and CS, contributing to improved mechanical and barrier properties (Amin et al., 2017).

### 3.10 Self-healing

**Fig. 6:**
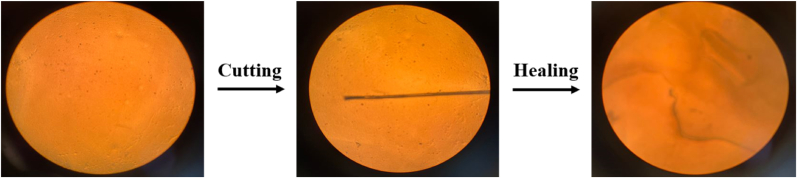
Self-healing ability of SCL/CS/CA cross-linked film. (SCL/CS/CA: cross-linked film based on secalin, corn starch, and citric acid).

Food packaging can be damaged at any stage from production to consumption throughout the distribution chain. The ability of packaging to self-heal is an important functional property. Materials with self-healing properties achieve this primarily through structural changes in the matrix and intermolecular interactions. During the healing process, specific non-covalent or covalent bonds first break and then recombine. Self-healing generally occurs in five steps: 1) polymer segments rearrange at the surface, 2) surfaces approach each other, 3) surfaces become wet, 4) inter-diffusion occurs between polymer chains, and 5) polymer chains undergo randomization. Several factors influence polymer rearrangement during self-healing, including molecular weight, surface roughness, and the nature of the polymer chains (Tavassoli et al., 2025). The results of the self-healing test for the SCL/CS/CA cross-linked film are shown in Fig. 6. The data indicate that the SCL/CS/CA cross-linked film possesses self-healing ability, which is a highly desirable property. After creating a scratch on the film and adding distilled water, the polymer chains recombined, and rearrangement on the film surface was observed within 10 min. Similar results were reported by Halloub et al. (2024), who developed a hemicellulose–chitosan film with good self-healing capability.

The self-healing efficiency of the selected cross-linked film was calculated to be 66.96 %, indicating that the prepared film possessed acceptable self-healing ability and was capable of partial recovery after damage, which could be beneficial during the distribution chain.

### 3.11 Mushroom packaging

Weight loss in fruits and vegetables during storage occurs as a result of water evaporation from the pericarp and cellular respiration. Mushrooms are classified as highly perishable vegetables due to their sensitivity to spoilage and high metabolic activity. Unlike many other vegetables, mushrooms lack a protective cuticle and have a thin epidermal structure, which makes them particularly prone to water loss and physical damage. In addition, their low ethylene production capacity combined with rapid respiration and transpiration further accelerates deterioration, browning, and microbial spoilage, resulting in a very short shelf-life (Shehata et al., 2020; Zhu et al., 2021). Therefore, developing methods to extend their shelf-life is critically important. One solution is the use of suitable packaging (Pérez-Bassart et al., 2023).

**Fig. 7:**
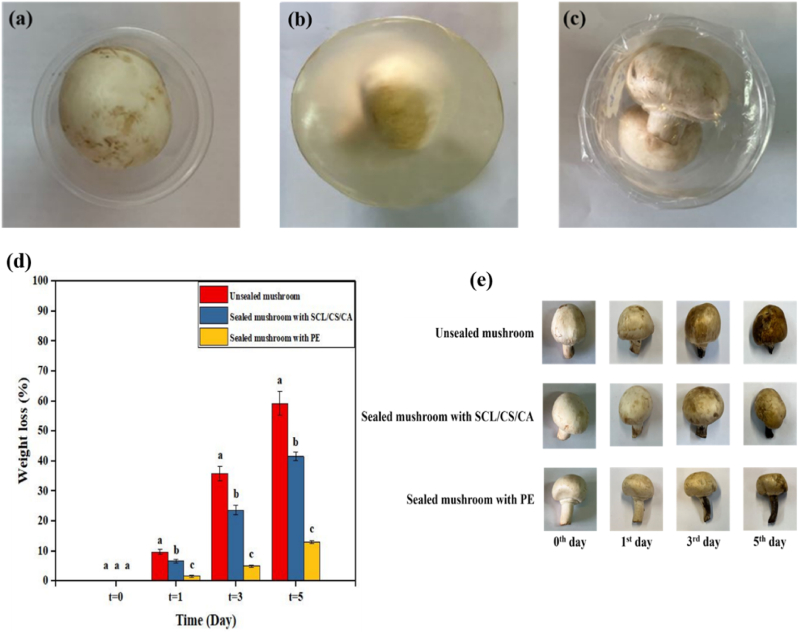
Mushroom storage in an unsealed dish (a), a dish sealed with the cross-linked film (b), a dish sealed with polyethylene (PE) film (c), weight loss (d) and appearance (e) of mushrooms during storage. (SCL/CS/CA: cross-linked film based on secalin, corn starch, and citric acid). Different letters denote significant differences at p < 0.05.

Additionally, the results for mushroom weight loss and appearance during storage are also presented in Fig. 7. Darkening and dehydration of the mushrooms began on the first day, with varying rates observed under different conditions. By the end of the 5th day, the unsealed mushrooms had darkened and dehydrated more severely, whereas those sealed with PE and SCL/CS/CA films exhibited slower deterioration in visual quality and reduced weight loss due to controlled permeability to water vapor and gases. In other words, by the end of the 5th day, the unsealed mushrooms were no longer of acceptable quality or appearance, whereas both PE and SCL/CS/CA cross-linked films maintained better physical appearance and freshness indicators. It should be noted that our assessment was limited to weight loss and visual attributes, and microbiological analyses were not performed; therefore, the observed improvements cannot be directly interpreted as extended microbiological shelf life. Wen et al. (2021) reported similar improvements in the shelf life of PVA/carboxymethyl chitosan (CMC)/CA-sealed cherry tomatoes and strawberries compared to unsealed samples.

## 4 Conclusion

In summary, blend films based on SCL and CS were successfully prepared, and CA was shown to be an effective cross-linker for improving their performance. The designed SCL/CS/CA cross-linked films exhibited enhanced physicochemical, barrier, and mechanical properties compared to the pure biopolymers. ATR-FTIR and TGA analyses confirmed good compatibility and interaction between SCL, CS, and CA. Among the tested formulations, the SCL/CS (2:1)/CA (2 %) film showed the most favorable combination of properties. The selected cross-linked film also demonstrated improved hydrophobicity, antioxidant capacity, and measurable antibacterial activity, particularly against Gram-negative bacteria. Furthermore, its application in mushroom packaging under ambient conditions provided preliminary evidence of potential usefulness in reducing quality deterioration. While further studies, especially on microbial spoilage and under commercial storage conditions, are needed, the results suggest that SCL/CS/CA films could be considered as promising candidates for biodegradable food packaging applications.

## CRediT authorship contribution statement

Sona Dodange: Conceptualization, Methodology, Formal analysis, Software, Data curation, Writing – original draft.

Hajar Shekarchizadeh: Conceptualization, Writing – review & editing, Validation, Investigation, Supervision.

## Declaration of competing interest

The authors declare the following financial interests/personal relationships which may be considered as potential competing interests: Sona Dodange reports financial support was provided by Iran National Science Foundation. If there are other authors, they declare that they have no known competing financial interests or personal relationships that could have appeared to influence the work reported in this paper.
